# Efficacy of Transcatheter Arterial Embolization for Hemorrhage Control in Traumatic Hepatic Injury With Celiac Axis Stenosis

**DOI:** 10.1155/emmi/5577388

**Published:** 2025-07-02

**Authors:** Yook Kim, Younghoon Sul

**Affiliations:** ^1^Department of Radiology, Chungbuk National University Hospital, Cheongju, Republic of Korea; ^2^Department of Radiology, Chungbuk National University College of Medicine, Cheongju, Republic of Korea; ^3^Department of Trauma Surgery, Chungbuk National University Hospital, Cheongju, Republic of Korea; ^4^Department of Trauma Surgery, Chungbuk National University College of Medicine, Cheongju, Republic of Korea

**Keywords:** celiac axis stenosis, hemorrhage control, transcatheter arterial embolization, traumatic liver injury, vascular anatomy

## Abstract

**Purpose:** This study aimed to evaluate the safety and efficacy of transcatheter arterial embolization (TAE) for hemorrhage control in patients with traumatic liver injury complicated by celiac axis stenosis (CAS).

**Methods:** Nine patients diagnosed with CAS who underwent TAE between January 2012 and December 2024 were included in this retrospective study. Imaging studies were used to assess the vascular anatomy, and clinical outcomes were analyzed, focusing on technical and clinical success rates and complications.

**Results:** All patients achieved technical success with TAE, with a clinical success rate of 77%, and no 30-day mortality. Seven patients had medial arcuate ligament compression, which was identified as the cause of CAS, while two patients had atherosclerosis.

**Conclusion:** TAE is a safe and effective intervention for managing hemorrhage in traumatic liver injury complicated by CAS. Thorough assessment of computed tomography images is crucial for diagnosing the underlying causes of CAS and optimizing catheterization strategies.

## 1. Introduction

Liver injuries constitute the most prevalent type of abdominal trauma, with an incidence of approximately 15%–20% [1]. Hemorrhage resulting from hepatic injury is a significant contributor to morbidity and mortality in patients with trauma [2]. Over the past 2 decades, there has been a paradigm shift toward nonoperative management for controlling hemorrhage following traumatic liver injury [3]. Transcatheter arterial embolization (TAE) has emerged as a prominent nonoperative strategy for blunt hepatic trauma with success rates between 85% and 100% [4]. A critical determinant of TAE success is the selective catheterization of the proper hepatic artery and its peripheral branches [5]. However, procedural challenges often arise due to unfavorable vascular anatomy, particularly in the context of celiac axis (CA) stenosis (CAS), which frequently precipitates the development of collateral pathways, such as the pancreaticoduodenal arcade [6]. While numerous studies have investigated interventional techniques for managing hepatic tumors, pancreaticobiliary surgery, and liver transplantation in patients with CAS, there is a paucity of literature specifically addressing the application of TAE for traumatic hepatic injuries in this cohort [7]. Therefore, this study aimed to retrospectively assess the safety and efficacy of TAE for hemorrhage control in patients presenting with blunt hepatic trauma complicated by underlying CAS.

## 2. Materials and Methods

**Figure 1:**
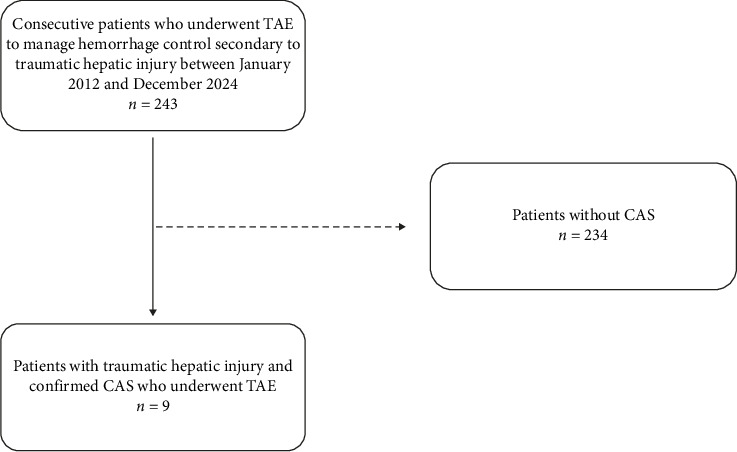
Flow diagram of patient selection. TAE, transcatheter arterial embolization; CAS, celiac axis stenosis.

This retrospective study was approved by the Institutional Review Board of the Chungbuk National University Hospital (date: November 2024, decision no: 2024-11-002), which waived the need for informed consent. Between January 2012 and December 2024, 243 patients were referred to our interventional unit for TAE to manage hemorrhage secondary to traumatic hepatic injury, as determined by clinical evaluation and computed tomography (CT) imaging. Among these patients, nine were diagnosed with CAS and subsequently underwent TAE for hepatic hemorrhage control (Figure 1).

Superior mesenteric arteriography revealed retrograde opacification of the CA and its branches, driven by collateral circulation from the superior mesenteric artery and distinct collateral pathways secondary to hepatic artery anatomical variants, indicating inadequate inflow from the celiac trunk. Traumatic hepatic hemorrhage was defined as the presence of contrast extravasation, intrahepatic hematoma, or hemoperitoneum on post-trauma CT scans.

Vital signs, including systolic and diastolic blood pressure and heart rate, along with laboratory parameters, such as hemoglobin levels, platelet counts, prothrombin time, activated partial thromboplastin time, and international normalized ratio, were assessed upon initial presentation in the clinic. The median values, along with the data range, were calculated using standard statistical methods. Moreover, the mechanism and classification of hepatic injury according to the American Association for the Surgery of Trauma (AAST), the Injury Severity Score (ISS), and CT findings of CAS and hepatic artery variations were collected and analyzed.

Details of the embolization procedures, including access routes via the CA or collateral vessels, procedure-related complications, and clinical outcomes following TAE (specifically technical success, clinical success, and 30-day mortality rate) were thoroughly evaluated.

### 2.1. Angiography and the Embolization Procedure

Angiography and embolization procedures were performed by two interventional radiologists, each with 6–10 years of expertise in endovascular treatments. After lidocaine administration, access was achieved via the right common femoral artery. Comprehensive celiac and superior mesenteric artery angiography was conducted to delineate the site of the hepatic artery injury using a 5F catheter (RH or RHR catheter, Cook Medical) and a 0.035-inch hydrophilic guidewire (Radifocus, Terumo Inc.).

Initial catheterization attempts directed at the hepatic artery utilized a combination of a 5-Fr catheter and microcatheter. In cases where traversal through an occluded CA proved impracticable, selective catheterization of the hepatic arteries was accomplished through dilated pancreaticoduodenal arcades. Superselection of the bleeding vessels was facilitated using a microcatheter ranging from 1.7 Fr to 2.2 Fr.

Once the hepatic arteries were successfully accessed via the microcatheter, embolization was performed with n-butyl-2-cyanoacrylate (Histoacryl, B. Braun) or gelatin sponge particles (Spongostan, Johnson & Johnson), depending on the nature of the hemorrhage and the operator's preference. Subsequent angiography was performed to confirm effective control of bleeding.

### 2.2. Definitions and Study Endpoints

The causes of CAS were categorized as follows: (1) medial arcuate ligament compression (MALC), diagnosed by direct visualization of the median arcuate ligament on CT, accompanied by characteristic superior notch formation evident on sagittal images and (2) the assessment of atherosclerosis of the CA, established when CT revealed atherosclerotic changes in the aorta, along with obstructive atheroma at the celiac orifice, with or without calcification.

Technical success was defined as successful superselective microcatheterization of the bleeding vessel and complete occlusion of the bleeding site, confirmed by angiography showing no contrast extravasation at the end of the embolization procedure.

Clinical success was defined as cessation of hemorrhage following TAE, as evidenced by the absence of recurrent bleeding or need for additional interventions during follow-up, without the need for re-embolization or surgical intervention.

Potential complications arising from the procedure were evaluated according to the Society of Interventional Radiology Standards of Practice Committee classification.

### 2.3. Statistical Analysis

This case series does not involve formal statistical analysis owing to its small sample size. Descriptive statistics were used to summarize patient demographics and clinical outcomes, which are presented as medians with ranges for continuous variables and frequencies with percentages for categorical variables. No statistical software or tests were utilized, and the statistical significance was not assessed.

## 3. Results

The characteristics and clinical outcomes of the nine patients who underwent TAE for hemorrhage control are summarized in Table 1. All patients presented with hepatic injury, hemoperitoneum, and contrast medium extravasation at the injury site on initial CT imaging. The median interval from the traumatic event to angiography was 9.2 h (range: 2–42 h). A short interval of less than 24 h was observed in eight patients (range: 2–10 h), while one patient experienced an extended interval of more than 24 h (42 h). The predominant mechanism of injury was motor vehicle collision (*n* = 5), followed by assaults (*n* = 2), pedestrian traffic accidents (*n* = 1), and traumatic fall (*n* = 1).

Three patients sustained additional injuries to intra-abdominal organs, including the kidneys, spleen, and adrenal glands. Extra-abdominal injuries were noted in six patients, specifically to the musculoskeletal system (*n* = 5/6), thoracic region (*n* = 6/6), and head and neck region (*n* = 3/6). Importantly, no active extravasation of contrast media was detected in the concomitantly injured organs during CT and angiographic evaluations.

Two patients exhibited a significant drop in blood pressure to below 90/60 mmHg; however, coagulopathy was not apparent. The median international normalized ratio across the cohort was 1.13 (range: 0.92–1.45), and the median platelet count was 224,000/μL (range: 107,000–433,000/μL). The median number of blood transfusions administered was 5.3 units of packed red blood cells (range: 0.0–22.0 units).

**Figure 2:**
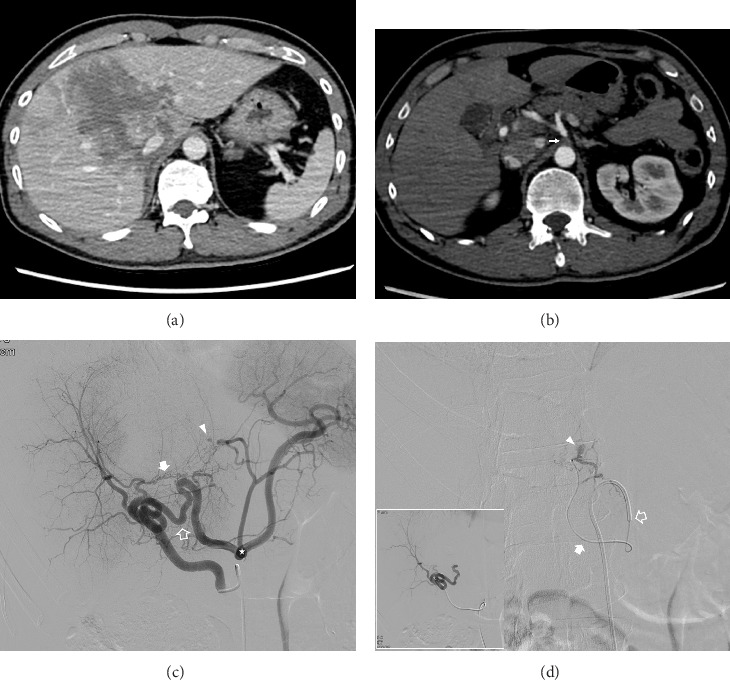
A 43-year-old male presented with blunt hepatic injury following a motorcycle accident. (a) Contrast-enhanced (CE) axial computed tomography (CT) during the portal phase reveals extensive ill-defined low-density lesions involving both lobes of the liver, indicative of blunt liver injury. (b) CE axial CT during the arterial phase demonstrates celiac axis stenosis (indicated by arrow) attributed to compression by the median arcuate ligament. (c) Selective angiography of the aberrant right hepatic artery originating from the superior mesenteric artery shows retrograde contrast material filling of the celiac branches (denoted by asterisk) through interlobar (open arrow) and intrahepatic (solid arrow) collateral vessels connecting the right and left hepatic arteries. Active hemorrhage (arrowhead) is also observed in segment IV of the liver. (d) Following the inability to advance the microcatheter to the site of bleeding through the intrahepatic collateral pathway (illustrated in the white-bordered box in the lower left), a decision was made to cannulate the stenotic segment of the celiac axis using a 5-Fr RHR catheter (open arrow). The microcatheter (solid arrow) was successfully advanced coaxially into the common hepatic artery and selective angiography of the hepatic artery revealed active bleeding (arrow), which was effectively managed with embolization using a 1:2 mixture of N-butyl cyanoacrylate and iodized oil.

**Figure 3:**
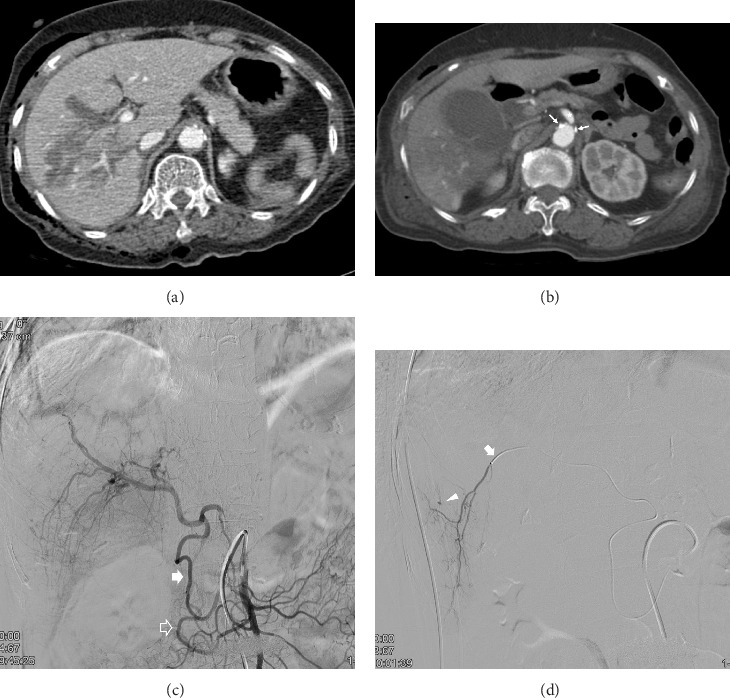
A 77-year-old female presented with blunt hepatic injury caused by a pedestrian traffic incident. (a) Contrast-enhanced (CE) axial CT during the portal phase demonstrates an irregular low-attenuation area suggestive of hepatic parenchymal laceration in the right lobe of the liver. (b) CE axial CT during the arterial phase indicates celiac axis stenosis (CAS) (represented by asterisk) with calcified plaques (arrows) at its origin. (c) Following unsuccessful attempts to cannulate the celiac axis (not depicted), superior mesenteric angiography reveals retrograde contrast material filling of the hepatic artery via the anterior (solid arrow) and posterior (open arrow) pancreaticoduodenal arcades, which originate separately from the superior mesenteric artery. (d) The microcatheter traversed the anterior pancreaticoduodenal arcade and successfully accessed the right hepatic artery (arrow). Selective angiography of the right hepatic artery demonstrated extravasation of contrast (arrowhead), which was successfully embolized using gelatin sponge particles (not shown).

Locations of the hepatic injuries, determined by CT imaging, were the right hemiliver (*n* = 4), left hemiliver (*n* = 2), and both lobes (*n* = 3). Imaging confirmed the presence of CAS in all patients at baseline. Among the identified causes of CAS, seven were attributable to MALC and two to atherosclerosis. Four patients had aberrant right hepatic arteries originating from the superior mesenteric artery. Direct catheterization of the CA was successfully achieved in seven patients with underlying MALC (Figure 2). In contrast, direct catheterization of the CA was unsuccessful in a patient with atherosclerosis-induced CAS, resulting in retrograde opacification of the hepatic arteries via the pancreaticoduodenal artery during superior mesenteric artery angiography (Figure 3). Therefore, selective hepatic angiography and subsequent embolization of the pancreaticoduodenal artery collateral vessels were performed in this case.

Embolization with a single agent (gelatine sponge particles) was used in six patients, whereas a combined approach using n-butyl-2-cyanoacrylate and gelatin sponge particles was employed in three patients. Technical success was achieved in all nine patients, with cessation of hemorrhage confirmed through complete angiography. Clinical success was attained in seven patients, all of whom were discharged without the need for further hepatic intervention, resulting in a clinical success rate of 77%.

Rebleeding occurred in two patients. One patient exhibited a decrease in hemoglobin levels (from 14.8 to 11.6) over a 24-h period, accompanied by an increased hematoma on a follow-up CT scan conducted 24 h after the initial TAE. This patient underwent repeat angiography, which revealed newly developed extravasation necessitating a second embolization with gelatin sponge particles, ultimately achieving successful hemorrhage control. The patient was discharged without further treatment of the hepatic injury. The second patient displayed an increased volume of hemoperitoneum without extravasation on a follow-up CT scan performed 2 days after the initial TAE, prompting surgical intervention (left hepatectomy) at the discretion of the surgical team. The patient was discharged without adverse events. Notably, the 30-day mortality rate of all patients in this case series was 0%.

## 4. Discussion

TAE via the CA has become increasingly prominent in the management of liver conditions including hemorrhage control, chemoembolization, radioembolization, and hepatic arterial infusion chemotherapy [8]. However, direct catheterization of hepatic arteries through the CA can be impeded by significant stenosis or occlusion. The reported incidence of CAS varies from 12.5% to 24% in Western populations, whereas in a Korean cohort of 400 patients, the prevalence of CA occlusion was 2.3%, with notable stenosis found in 7.3% of cases (29/400 patients) [5, 8]. The etiology of CAS includes atherosclerosis, pancreatitis, tumor invasion, agenesis, iatrogenic arterial injury during catheterization or surgical procedures, and extrinsic compression by the median arcuate ligament [9]. Among these factors, atherosclerosis and extrinsic MALC are the primary contributors. The literature indicates that atherosclerosis is the predominant cause of celiac stenosis in Western populations [5]. Conversely, in a study involving a Korean cohort with hepatocellular carcinoma, MALC was found in 55% of patients with celiac stenosis [7]. CT findings that suggest celiac compression by the median arcuate ligament typically include effacement or narrowing of the CA, dilated peripancreatic collateral vessels, and poststenotic dilation of the distal CA [6]. In this study, the presence and etiology of CAS were evaluated using CT imaging prior to angiography, confirming MALC in five patients (83.3%) and atherosclerosis in one patient (16.7%) [10]. The high prevalence of MALC may be attributed to the racial composition of the study cohort, which exclusively included Korean participants.

Pancreaticoduodenal arcades are crucial collateral vessels in patients with CAS. These vessels are generally not visualized in the absence of CA or superior mesenteric artery stenosis [11]. In circumstances of occlusion or substantial stenosis of the CA, the hepatic blood supply is predominantly derived from a retrograde arterial flow through the pancreaticoduodenal arcades, which can lead to arterial enlargement, rendering the pancreaticoduodenal arteries more prominent in superior mesenteric artery angiograms [6]. Given their utility as potential access routes for hepatic embolization, anatomical variability in the hepatic artery origin can result in atypical collateral pathways, as demonstrated in our patient cohort [12].

Two strategies may be employed to navigate the CAS: direct catheterization of the CA or alternative access via collateral pathways [6]. Direct catheterization offers the advantages of reduced procedural duration and facilitates effective manipulation. Nevertheless, inexperienced angiographers may encounter an elevated risk of arterial dissection due to repeated catheterization attempts, particularly when the celiac trunk is significantly compressed or occluded. If direct celiac catheterization fails, the utilization of collateral pathways is imperative. The inability to advance the microcatheter past the right hepatic artery is a significant challenge, as it hampers the achievement of superselective catheterization of distal bleeding vessels and is primarily related to the loss of torquability and pushability of the microcatheter when traversing the tortuous pathway through the pancreaticoduodenal artery [12]. According to Kwon et al. [7], compared to atherosclerotic occlusion, extrinsic compression from the median arcuate ligament may preserve the lumen of the CA. To minimize procedural duration and mitigate risks associated with ineffective manipulation, direct hepatic artery access through the CA should be the initial approach whenever feasible, particularly in cases of MALC. Recent advances in the management of challenging vascular anatomies, particularly in scenarios involving anatomical variants such as CAS, have been extensively discussed in the recent literature. These studies highlight the ongoing challenges and innovations pertinent to embolization strategies, emphasizing the importance of tailored approaches to improve technical success in complex settings [13, 14]. In this study, direct celiac catheterization was attempted in all patients, resulting in successful TAE in five patients with MALC. In contrast, one patient with atherosclerotic stenosis experienced a failure of direct catheterization, necessitating successful access through the PDA for embolization [10].

This study has some inherent limitations. First, it adopted a nonrandomized, retrospective design based on a review of medical records; prospective, randomized clinical studies could provide more definitive evidence regarding the efficacy and safety of the intervention. Second, the findings may not be directly applicable to Western populations because of the small sample size of the Korean cohort in which extrinsic compression by the median arcuate ligament is predominantly recognized as the cause of celiac occlusion. Additionally, the limited sample size reduces the statistical power to detect small effect sizes and may influence the stability of our estimates.

TAE is a safe and effective intervention for patients with traumatic liver injuries complicated by hemorrhage and CAS. To improve the success rate of TAE, it is imperative to meticulously assess CT images to accurately delineate the underlying causes of CAS. Additionally, catheterization strategies should prioritize access via the occluded CA, particularly in cases in which CAS is attributable to MALC.

In summary, this study provides valuable insights into the management of hemorrhage in traumatic liver injury complicated by CAS, addressing a critical gap in the current interventional radiology practices. Our findings could inform procedural strategies in similar clinical scenarios, ultimately improving patient outcomes.

## Figures and Tables

**Table 1:** Patient characteristics and clinical outcomes.

No./sex/age (y)	Trauma type	Interval^∗^(hours)	AAST Gr.	Injured area	CAS cause	Concomitant hepatic artery variation	Access route	Technical success	Clinical success	Remark
1/M/27	MVC	42	4	S8	MALC	None	Celiac axis	Yes	Yes	
2/M/56	MVC	8	4	S1, 4	MALC	None	Celiac axis	Yes	Yes	
3/F/54	Pedestrian TA	3	3	S4	MALC	None	Celiac axis	Yes	Yes	
4/F/77	Assault	9	3	S5	ASO	None	PDA	Yes	Yes	
5/M/43	MVC	2	3	S1, 4, 8	MALC	RHA from SMA	Celiac axis, SMA	Yes	No	Rebleeding ⟶ 2nd embolization
6/F/55	MVC	3	4	S2, 4, 8	MALC	RHA from SMA	Celiac axis, SMA	Yes	No	Exploratory laparotomy due to increased amount of hemoperitoneum on following CT scan after 2 days from TAE
7/M/64	MVC	3	4	S8	MALC	None	Celiac axis	Yes	Yes	
8/M/69	Fall down	3	4	S8	ASO	RHA from SMA	SMA	Yes	Yes	
9/M/53	Assault	10	5	S4, 5, 8	MALC	RHA from SMA	Celiac axis, SMA	Yes	Yes	

*Note:* ASO, atherosclerosis.

Abbreviations: MALC, median arcuate ligament compression; MVC, motor vehicle collision; PDA, pancreaticoduodenal arcade; RHA, right hepatic artery; SMA, superior mesenteric artery; TA, traffic accident.

^∗^Interval indicates the time interval between trauma and angiography.

## Data Availability

The data that support the findings of this study are available from the corresponding author upon reasonable request.
